# Engineering the MoO*_x_*/CIGS Interface for Enhanced Performance and Suppressed Recombination in Industrial Modules

**DOI:** 10.3390/ma18245569

**Published:** 2025-12-11

**Authors:** Mingguang Chen, Yao Gao, Bitao Chen, Disheng Yao, Guoyuan Zheng, Jilin Wang, Shuyi Mo, Yong Peng, Fei Long

**Affiliations:** 1 Guangxi Key Laboratory of Optical and Electronic Material and Devices, School of Materials Science and Engineering, Guilin University of Technology, Jiangan Road 12, Guilin 541004, China; 2 Collaborative Innovation Center for Exploration of Nonferrous Metal Deposits and Efficient Utilization of Resources, Guilin University of Technology, Jiangan Road 12, Guilin 541004, China; 3 State Key Lab of Advanced Technology for Materials Synthesis and Processing, Wuhan University of Technology, Wuhan 430070, China

**Keywords:** CIGS solar cells, MoO*_x_* interlayer, back contact passivation, MoSe_2_ thickness reduction, interfacial engineering

## Abstract

**Highlights:**

**What are the main findings?**

- An in-situ and controllable reactive sputtering technique is applied for MoO*_x_* deposition.
- MoO*_x_* incorporation reduces voids and MoSe_2_ thickness by suppressing the Se diffusion.
- MoO*_x_*-incorporated CIGS achieves higher PV performance by forming a favorable band alignment.
- MoO*_x_* interfacial layer can improve the PV performance of the large-scale (1650 × 658 mm^2^) module.

**What are the implications of the main findings?**

- Provides a viable, industry-compatible approach to enhance CIGS module performance.
- Offers a scalable method to suppress MoSe_2_ formation and improve interfacial adhesion.
- Demonstrates effective rear-interface passivation and band engineering for higher efficiency.
- Supports the development of high-efficiency, large-area CIGS solar modules for commercial use.

**Abstract:**

This study investigates how molybdenum oxide (MoO*_x_*) rear interface passivation—specifically its thickness and deposition conditions—affects CIGS thin-film solar cells. The MoO*_x_* layer effectively suppresses selenium/sulfur diffusion into the molybdenum back contact during high-temperature processing, while improving the absorber’s microstructure by reducing interfacial voids. These modifications enhance electrical properties, yielding lower series resistance, higher shunt resistance, and improved fill factor and current density. Although recombination increases slightly, the reduction in voltage-related fill factor loss ultimately boosts hole extraction and suppresses electron recombination at the back contact. Consequently, MoO*_x_*-passivated cells achieve superior performance, with industrial-scale modules (1650 mm × 658 mm) reaching 152.41 W output power and 14.0% efficiency. This work provides valuable insights for optimizing MoO*_x_*-based interface engineering to improve CIGS solar cell efficiency and manufacturability.

## 1. Introduction

Copper indium gallium selenide (CIGS) is recognized as one of the most promising solar cell materials, which achieves a record efficiency of 23.64% by implementing a rubidium fluoride post-deposition treatment on silver-doped Cu(In,Ga)Se_2_ (ACIGS) absorbers [1]. CIGS exhibits high power conversion efficiency, a good absorption coefficient, superior long-term stability, and a relatively short energy payback time [2,3]. Various methods have been utilized for the fabrication of high-quality CIGS absorbers; however, rear interface recombination hinders further efficiency improvement. Therefore, passivating the rear interface of the CIGS absorber can reduce recombination and improve the photovoltaic (PV) performance of CIGS solar cells.

Molybdenum (Mo) is employed as the back contact for the CIGS solar cells. When the precursor thin films are subject to selenization in a high selenium concentration atmosphere, a large-grained and high-quality CIGS absorber with significantly low selenium vacancies can be obtained. In addition, a thin MoSe_2_ interlayer spontaneously forms at the Mo/CIGS interface during high-temperature processing, achieving a favorable quasi-ohmic MoSe_2_/CIGS contact and enhancing hole extraction [4]. However, the Mo back contact exhibits strong parasitic absorption, leading to severe optical losses. It is reported that a cliff-like band alignment forms at the MoSe_2_/CIGS interface, introducing a high hole transport barrier (*Φ_B_* = 340 mV) [5]. Furthermore, when the MoSe_2_ layer becomes thicker than a certain threshold (≥100 nm), it increases the series resistance (R_s_) of the device, resulting in low PV performance. Additionally, the as-formed thick MoSe_2_ layer leads to pronounced lattice expansion, degrading the adhesion at the MoSe_2_/CIGS interface [6]. Therefore, developing effective strategies to reduce the thickness of the MoSe_2_ layer presents a viable route to improving the efficiency of CIGS solar cells.

The MoO*_x_* layer has been utilized for selecting photo-generated holes from silicon heterojunction (SHJ) solar cells, thereby improving the efficiency [7]. An ultra-thin MoO*_x_* layer (~10 nm) with a high work function (~5.9 eV) and a wide bandgap (~2.9 eV) has been introduced to the Mo/CIGS interface for rear interface passivation and band alignment adjustment [8]. The ultra-thin MoO*_x_* interfacial layer significantly reduces carrier recombination and suppresses the formation of a thick MoSe_2_ layer, leading to enhanced open-circuit voltage (*V_oc_*) and fill factor (*FF*) [9,10,11]. Adjustment of the oxygen vacancy concentration of the MoO*_x_* layer can optimize electrical conductivity and work function, thereby modulating the interfacial transport barrier and the series resistance of the device [8,12]. Meanwhile, a thin MoO*_x_* layer can suppress the formation of MoSe_2_ at the Mo/CIGS interface [5], offering a pathway for interface engineering.

Li et al.’s simulation results demonstrate that a thin MoO*_x_* layer not only suppresses the formation of MoSe_2_ at the Mo/CIGS interface but also reduces the back-contact barrier and induces the formation of a “spike-like” band alignment [5]. As a result, holes can be extracted efficiently by the back contact, while electrons are repelled toward the front interface, thereby reducing recombination at the rear interface of the CIGS absorber layer [5]. He et al. reported that the deposition of the MoO*_x_* layer on the rear interface of the ultra-thin CIGS devices reduced the back barrier height from 43.8 meV to 16.0 meV [10]. Meanwhile, Zeng et al. fabricated a MoO*_x_* via oxygen plasma treatment on the Mo back contact, which acts as a rear interface passivation layer for effectively suppressing recombination at the rear interface of CIGS and leading to notable improvements in both open-circuit voltage (*V_oc_*) and fill factor (*FF*) [9]. Given that the work function of MoO*_x_* is thickness-dependent, the thickness of the MoO*_x_* layer influences the band alignment and back-barrier properties at the rear interface of the CIGS absorber. Therefore, optimizing the MoO*_x_* thickness is essential for enhancing the performance of CIGS thin-film solar cells.

A thin MoO*_x_* rear interface passivation layer has been shown to improve the performance of the lab-scale CIGS solar cells; however, systematic studies on how the MoO*_x_* layer affects the performance of CIGS modules remain limited. In this work, through multiple experimental validations, we systematically investigate the interfacial passivation behavior of MoO*_x_* layers with various fabrication parameters (absorber annealing atmospheres, MoO*_x_* thickness, and the O_2_ flow rate during the MoO*_x_* deposition). By combining current-voltage (*J-V*) characterization, external quantum efficiency (EQE) analysis, and interfacial microstructure characterization, we establish a quantitative correlation between the MoO*_x_* passivation layer and key photovoltaic parameters (*V_oc_*, *FF*, *R_s_*). The quantitative analysis of non-radiative and radiative recombination is utilized to investigate the mechanism of the PV performance improvement by introducing MoO*_x_* passivation for the CIGS solar cells. Furthermore, the figures of merit (FoM) based on the Shockley–Queisser (SQ) model were employed to investigate the dominant factor for the enhanced photovoltaic performance of the MoO*_x_*-passivated CIGS devices. Finally, the MoO*_x_* interface passivation layer is utilized for large-area CIGS modules (1650 mm × 658 mm).

## 2. Materials and Methods

### 2.1. Device Fabrication

Soda-lime glass (SLG) substrate (1650 mm × 658 mm × 2 mm) was sequentially cleaned with acetone, iso-propanol, and deionized water for 15 min each by sonication. A 50 nm SiO*_x_* layer was sputtered on the as-cleaned SLG in an Ar/O_2_ mixed atmosphere at 1.5 Pa to hinder the Na diffusion from the SLG substrate during the high-temperature selenization process. Subsequently, a MoO*_x_* layer (45–60 nm thick) was deposited on the SiO*_x_*-coated SLG substrate via sputtering in an Ar/O_2_ mixed atmosphere to address the thermal expansion coefficient mismatch between SLG and metallic Mo for improving the adhesion. Following this, a 600 nm-thick metallic Mo layer was then deposited by DC sputtering under pure Ar gas at 1.5 Pa for the formation of an SLG/SiO*_x_*/MoO*_x_*/Mo back contact (resistivity less than 2.3 × 10^−5^ Ω·cm). 

Various thicknesses of MoO*_x_* passivation layers (10–20 nm) were sputtered onto the surface of the SLG/SiO*_x_*/MoO*_x_*/Mo stack. The prepared Mo back electrode with and without the MoO*_x_* passivation layer was left at atmospheric pressure for 6–8 h to release thin-film stress before being segmented into 141 Mo sub-electrodes using laser etching (P1). Subsequently, CuGa:Na (CGN), CuGa (CG), and In layers were sputtered onto the back electrode (with/without MoO*_x_*) to form a CIG metallic precursor multilayer structure, namely 50 nm CGN/380 nm CG/450 nm In.

The deposited CIG precursor films were selenized in an H_2_Se atmosphere at 450 °C. Residual H_2_Se was then evacuated, and H_2_S gas was introduced for sulfurization at 540 °C. After the sulfurization-after-selenization (SAS) annealing, the chamber was allowed to cool naturally. A CdS layer (20–40 nm thick) was deposited by chemical bath deposition (CBD) using an aqueous solution containing 0.015 M cadmium sulfate, 0.050 M thiourea, and 0.65 M ammonia water.

Following the P2 etching process, the samples were transferred to a metal–organic chemical vapor deposition (MOCVD) system for the sequential deposition of a 50 nm thick i-ZnO layer and a 1500–1700 nm thick B-doped ZnO layer. After device assembly, the P3 etching and P4 edge isolation processes were performed to ensure edge insulation. The large-area CIGS devices were then divided into small cells with an area of 0.43 cm^2^ via glass scribing. Positive and negative electrodes were finally drawn from adjacent Mo layers using indium bonding for subsequent external quantum efficiency (EQE) testing or other performance characterizations.

### 2.2. Characterization

The surface and cross-sectional morphologies of the films were characterized by a thermal field emission scanning electron microscope (FESEM, GeminiSEM 500, Carl Zeiss, Oberkochen, Germany). The CIGS layer was selectively etched using a 1% bromine in methanol solution (by volume). This solution is a well-established etchant for chalcopyrite-based absorbers and allows for the complete removal of CIGS without significantly attacking the underlying Mo-based back contact. The samples were immersed in the etching solution for approximately 30–60 s under gentle agitation, followed by thorough rinsing with methanol and drying under a nitrogen stream. This process effectively exposes the morphology of the back contact interface, enabling clear observation of the Mo(S,Se)_2_ nanostructures in the reference sample and the preserved Mo morphology in the MoO*_x_* -passivated sample. The phases and crystal structures were identified by XRD (X’Pert PRO, Malvern PANalytical, Malvern, UK) using a monochromatic Cu K_α_ (λ = 0.154 nm) excitation source with 40 kV and 40 mA. Before the IV measurement, the CIGS solar cells underwent a 20-min light-soaking process, and the IV performance of the 141 series-connected devices was tested. The photovoltaic performance of the CIGS module was tested by the GIV-20 series tester (Gsola Company Co., Ltd., Xi’an, China) with AAA solar simulators (Gsola Company Co., Ltd., Xi’an, China). The photovoltaic performance of the CIGSSe solar cells was measured with an AAA solar simulator (XES-301S, SAN-EI Electric Co., Ltd., Osaka, Japan) under AM 1.5 G irradiation with an intensity of 100 mW/cm^2^. The current density-voltage (*J-V*) curve was obtained by a Keithley 2400 Source Meter (Keithley 2400, Tektronix, Beaverton, OR, USA). External quantum efficiency (EQE) spectra were collected at the DC mode by a monochromatic incident photon-to-electron conversion efficiency (IPCE) system (Solar Cell Scan 100, Zolix instruments. Co., Ltd., Beijing, China).

## 3. Results and Discussion

**Figure 1:**
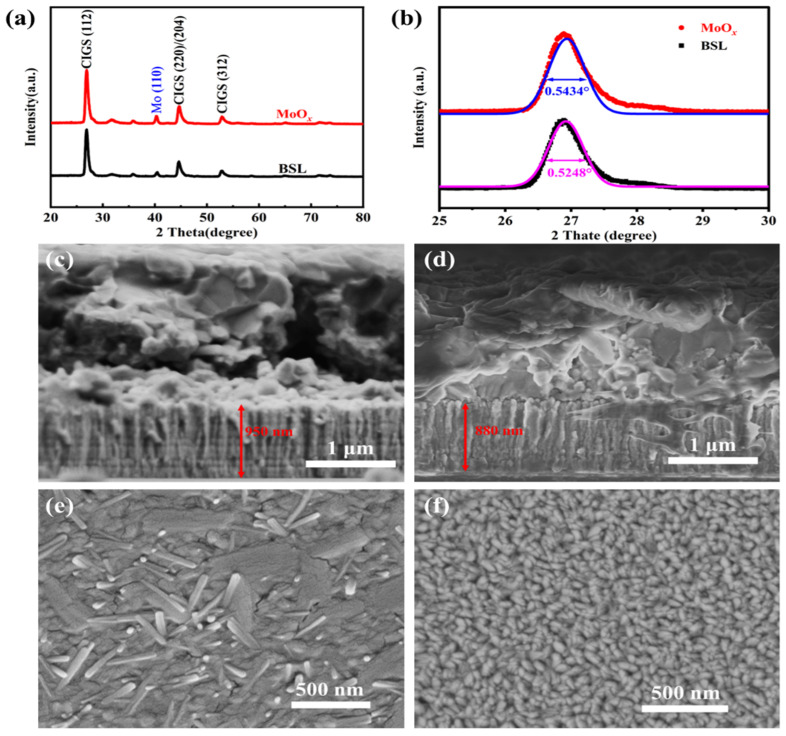
The X-ray diffraction (GIXRD) patterns: (**a**) Comparison GIXRD pattern of the CIGS absorption layer with a fixed 4.5° grazing incidence angle, (**b**) comparison GIXRD pattern of the CIGS absorption layer with a fixed 0.5° grazing incidence angle (pink fitting: BSL and blue fitting: MoO*_x_*). Cross-sectional SEM images of (**c**) without and (**d**) with MoO*_x_* passivated CIGS absorbers. Top-view SEM images of removing the CIGS absorber to release the back electrode: (**e**) without and (**f**) with MoO*_x_* for interfacial passivation.

The grazing-incidence X-ray diffraction (GIXRD) patterns of the CIGS samples with/without a MoO*_x_* passivation layer exhibit similar Bragg reflections, as shown in Figure 1a. The strongest Bragg reflection is located at 27.05°, corresponding to the (112) plane of the CIGS absorber layer [4]. Additionally, Bragg reflections belonging to the (220)/(204) and (312) planes of the CIGS absorbers are also observed from the GIXRD patterns. Furthermore, the Bragg reflection belonging to the Mo back contact was also observed at 40.4º. In the XRD pattern shown in Figure 1a, the variation in the peak intensity of Mo (110) (2θ = 40.4°) reflects differences in the relative content of Mo. A higher Mo peak intensity indicates that less Mo has been selenized into MoSe_2_, which enhances the adhesion between CIGS and Mo and thereby reduces voids at the CIGS–Mo interface. The full width at half maximum (FWHM) results in Figure 1b suggest a slight decrease in the crystallinity of CIGS, which is likely attributed to the hindered diffusion of Na after the introduction of the MoO*_x_* layer. The enlarged GIXRD of the patterns in the 25–30° range (Figure 1b) reveals that the MoO*_x_*-passivated CIGS displayed a broader full width at half maximum (FWHM) of the (112) Bragg reflection than without MoO*_x_*, indicating a reduction in the grain size of the CIGS absorber layer after the introduction of MoO*_x_*. It is reported that gallium (Ga) and oxygen (O) exhibit high chemical reactivity; GaO*_x_* is observed from the interface after sputtering a transparent conductive oxide onto the CIGS surface at room temperature [13]. Furthermore, when sodium (Na) is present in the precursor film (CuGa:Na layer), it can catalyze the reaction between Ga and O at the high-temperature selenization process, leading to the formation of a thicker GaO*_x_* layer [14]. Therefore, the reduction in grain size of MoO*_x_*-passivated CIGS absorber can be attributed to the reaction between oxygen species from MoO*_x_* and Ga within the absorber layer.

Figure 1c,d present cross-sectional scanning electron microscopy (SEM) images of the Mo/CIGS back-contact multilayer structure without and with the MoO*_x_* passivation layer, respectively. After the introduction of a MoO*_x_* for rear interface passivation, the back contact thickness is reduced to 880 nm (Figure 1d) from 950 nm for the case without MoO*_x_* passivation (Figure 1c). A thinner back contact layer of the MoO*_x_*-passivated CIGS can be explained by the suppression of Se diffusion during the selenization process, which effectively inhibits the formation of a MoSe_2_ interfacial layer. Furthermore, the CIGS layer in the MoO*_x_*-passivated sample displays an improved microstructure with significantly fewer voids at the rear interface, indicating that the introduction of MoO*_x_* is beneficial to the elimination of the interfacial issues caused by the thermal expansion coefficient mismatch between the CIGS absorber and the back contact electrode.

Top-view SEM images of the back contact surfaces after removal of the CIGS absorber layer are present in Figure 1e,f. The needle-like or rod-like Mo(S,Se)_2_ nanoparticles are observed from the CIGS without a MoO*_x_* passivation layer (Figure 1e), indicating that the Mo reacted with Se and S during the high-temperature annealing process. In contrast, when a thin MoO*_x_* layer was deposited on top of the Mo back electrode (Figure 1f), the morphology of Mo was retained, hindering the formation of Mo(S,Se)_2_ nanostructures. This result further confirms that the MoO*_x_* passivation layer can effectively block the diffusion of Se and S toward the Mo back electrode during the high-temperature SAS process.

**Figure 2:**
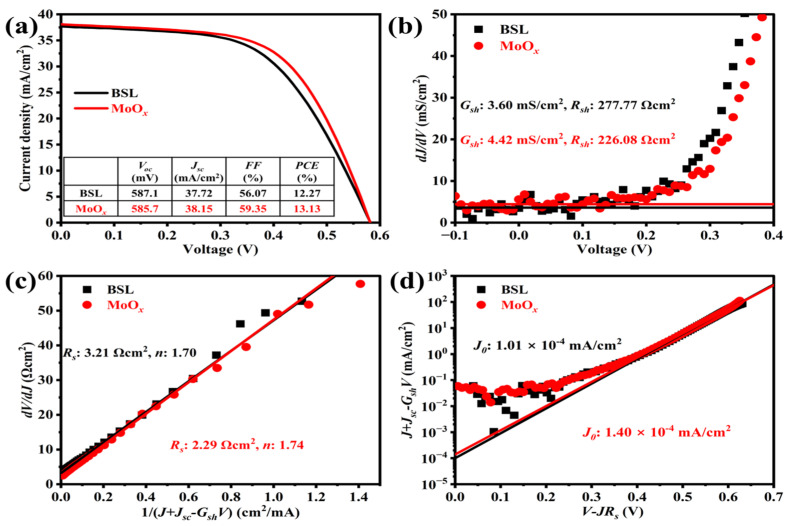
Photovoltaic and electrical parameters of CIGS solar cells without (black) and with (red) the MoO*_x_* rear interface passivation layer: (**a**) J-V curves, (**b**) shunt conductivity results, (**c**) series resistance results, and (**d**) reverse saturation current density.

The current density-voltage (*J-V*) characteristics of CIGS solar cells with and without the MoO*_x_* rear interface passivation layer are shown in Figure 2a. The MoO*_x_*-passivated CIGS presents a higher power conversion efficiency (*PCE* = 13.13%) than the reference one (*PCE* = 12.27%), which can be explained by the increase in both short-circuit current density (*J_sc_*) and fill factor (*FF*). The improvements in *J_sc_* and *FF* can be ascribed to the higher work function of MoO*_x_* than MoSe_2_ and Mo [5], which promotes favorable energy level alignment with the CIGS absorber layer [5,10]. This band alignment reduces the hole transport barrier at the rear interface and enhances hole extraction efficiency toward the back electrode [10]. Moreover, due to the similar device structure and Ga/(Ga+In) (GGI) ratio of the absorber layer in both types of cells—with the only difference being the thin MoO*_x_* modification at the rear interface—their open-circuit voltage (*V_oc_*) values remain comparable.

In terms of electrical parameters, the MoO*_x_*-passivated device exhibits a slightly higher shunt conductance (*G_sh_* = 4.42 mS/cm^2^) than the reference device (*G_sh_* = 3.60 mS/cm^2^) (as shown in Figure 2b), which may be associated with an increase in the surface roughness of the MoO*_x_*-passivated CIGS (Figure 1e). Concurrently, after introducing the MoO*_x_* layer, the series resistance decreased from 3.21 Ω·cm^2^ to 2.29 Ω·cm^2^, while the ideality factor increased slightly from 1.70 to 1.74 (Figure 2c). These changes indicate more efficient extraction of photogenerated carriers, thereby contributing to the higher *J_sc_* observed in Figure 2a. However, likely due to the slightly reduced crystallinity of the CIGS absorber after MoO*_x_* passivation (Figure 1b), the reverse saturation current density (*J*_0_) also increased to some extent (Figure 2d).

**Figure 3:**
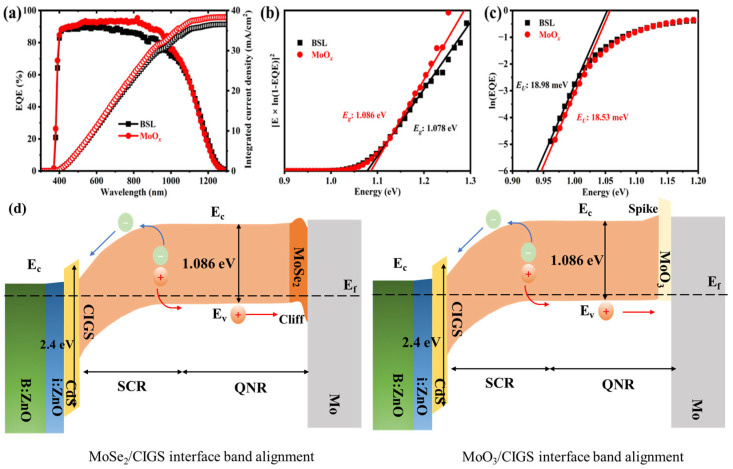
(**a**) EQE spectra and integrated current density, (**b**) band gap energies, (**c**) Urbach energies of the CIGS devices with (red line) and without MoO*_x_* (black line) for rear interface modification, and (**d**) diagram of the band alignment between the CIGS and Mo layers, inserting MoSe_2_ and MoO_3_, respectively. (Green is B:ZnO layer, blue is i-ZnO, yellow is CdS, light orang is CIGS, dark orang is MoSe_2_, light yellow is MoO_3_).

In the wavelength range of 350–420 nm, an overlap in the external quantum efficiency (EQE) spectral response is observed, which is attributed to the optical absorption of the i-ZnO and B:ZnO window layers. Compared to the reference CIGS, the MoO*_x_*-passivated CIGS cell exhibits a higher EQE response across the 450–1050 nm spectral range (Figure 3a). This enhancement can be correlated with the reduced void density at the rear interface region (as evidenced by the cross-sectional morphology in Figure 1d). The improved microstructure of the rear interface is favorable to reducing parasitic absorption from the Mo back electrode, thereby enhancing the photocurrent contribution in this spectral region. Owing to the superior EQE response in the visible to near-infrared range, the MoO*_x_*-passivated CIGS achieves a higher integrated current density (Figure 3a), which is in good agreement with the *J-V* measurement results (Figure 2a).

The bandgap energy of the absorber layer, derived from the EQE spectra, is determined to be 1.086 eV for the MoO*_x_*-passivated CIGS, slightly larger than the 1.078 eV of the reference sample (Figure 3b). The slightly higher bandgap may be attributed to the fact that the introduction of the MoO*_x_* layer suppresses the reaction between sulfur and the Mo back electrode during the post-sulfurization process. As a result, more sulfur participates in the reaction within the CIGS absorber layer, partially substituting Se in the lattice or passivating selenium vacancies, leading to an increased sulfur content in the absorber and a slight widening of the bandgap. Furthermore, the MoO*_x_*-passivated CIGS exhibits a reduction in the Urbach energy (Figure 3c), indicating that the rear interface passivation does not degrade the crystalline quality of the CIGS absorber.

The most critical electronic effect of replacing the native MoSe_2_ with a MoO_3_ interlayer is the modification of the energy band alignment at the back contact. This is schematically illustrated in Figure 3d, where the reference MoSe_2_/CIGS interface, a “cliff-like” valence band offset, is typically formed. This alignment creates a significant energy barrier for hole transport from the CIGS valence band into the back contact, while failing to effectively block electrons, leading to pronounced interface recombination. In contrast, the MoO_3_/CIGS interface facilitates a “spike-like” band alignment. The high work function (~5.9 eV) and wide bandgap (~2.9 eV) of MoO_3_ cause a strong upward band bending in the CIGS absorber near the interface. This creates a beneficial energy spike in the conduction band, which efficiently blocks electrons from reaching the back contact, redirecting them toward the front junction. Simultaneously, the alignment creates a near-ideal, low-barrier pathway for hole extraction from the CIGS valence band into the MoO_3_ layer and subsequently to the Mo electrode. Therefore, the MoO_3_ interlayer serves not merely as a passive barrier but as an active interface engineer.

Based on the principles of black-body radiation theory and external quantum efficiency (EQE) data, the radiative voltage (*V_oc,rad_*) can be calculated from the radiative saturation current density (*J*_0*,rad*_) and integrated current density from the EQE responses of the solar cell. Combining the *V_oc,SQ_*, *V_oc,rad_*, and *V_oc_* can quantitatively obtain the contributions of radiative recombination losses, non-radiative recombination losses, and total recombination losses. Furthermore, applying the detailed balance theory enables the corresponding physical loss mechanisms analysis of solar cells based on the figures of merit (FoM).

The radiative voltage can be calculated using the following Equation [15,16]:

(1) $$V_{oc,rad}=\frac{k_{B}T}{q}\ln\left(\frac{J_{sc}}{J_{0,rad}}+1\right)$$

where *J*_0*,rad*_ is the radiative saturation current density and *J_sc_* is the integrated current density of the device. *J*_sc_ can be calculated by the following Equation [15,17]:

(2) $$J_{sc}=q\int_{0}^{\infty }EQE\left(E\right)\cdot \phi_{sun}\left(E\right)dE$$

where $\;\phi_{sun}\left(E\right)$ is the photon flux of the AM1.5G solar spectrum. *J*_0*,rad*_ can be calculated by the following Equation [15,16]:

(3) $$J_{0,rad}=q\int_{0}^{\infty }EQE\left(E\right)\cdot \phi_{BB}\left(E\right)dE$$

where $\phi_{BB}\left(E\right)$ is the photon flux of the black-body radiation spectrum:

(4) $$\phi_{BB}=\frac{2\pi }{c^{2}h^{3}}\frac{E^{2}}{\left[exp\left(\frac{E}{k_{B}T}\right)-1\right]}$$

Here, *h* is Planck’s constant and *c* is the speed of light in vacuum. Therefore, *ϕ_BB_* is a function of temperature; increasing the temperature leads to a higher photon flux from the black-body radiator.

Total voltage loss:

(5) $$Total\;V_{oc}\;losses=V_{oc,SQ}-V_{oc}$$

Radiative recombination voltage loss:

(6) $$Radiative\;V_{oc}\;losses=V_{oc,SQ}-V_{oc,rad}$$

Non-radiative recombination voltage loss:

(7) $$Nonradiative\;V_{oc}\;losses=V_{oc,rad}-V_{oc}$$

**Figure 4:**
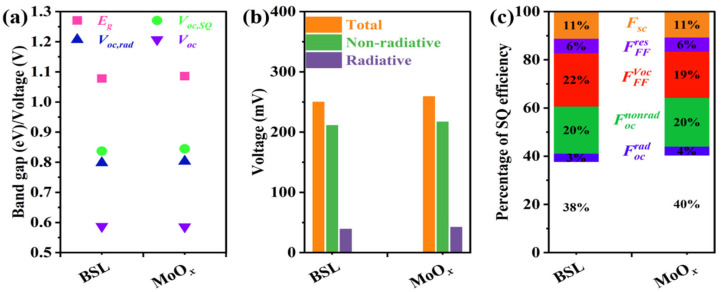
Recombination analysis of the CIGS solar cells without/with MoO*_x_* for rear interface modification: (**a**) band gap energy, *V_oc,SQ_*, *V_oc,rad_*, and *V_oc_*, (**b**) total *V_oc_* losses, non-radiative *V_oc_* losses, and radiative *V_oc_* losses extracted from the *J-V* and EQE results; and (**c**) figures of merit (FoM) based on the SQ model equations.

After incorporating the MoO*_x_* passivation layer, the bandgap of the CIGS absorber increased by 8 meV (Figure 3b and Table 1), leading to a corresponding rise in its Shockley–Queisser (SQ) theoretical limit open-circuit voltage (*V_oc,SQ_*) from 837.0 mV to 844.5 mV (Figure 4a and Table 1). Benefiting from the superior spectral EQE response, the MoO*_x_*-modified device achieved a radiative voltage (*V_oc,rad_*) of 802.8 mV, representing a 4.4 mV enhancement over the reference sample. Consequently, based on the black-body radiation theory and utilizing the measured *V_oc_* from the *J-V* characterization (Figure 2a) alongside the EQE data, the total open-circuit voltage loss can be calculated according to the theoretical calculation equation. These results indicate that the total open-circuit voltage loss of the MoO*_x_*-passivated CIGS increased to 258.8 mV from 249.9 mV for reference CIGS (Figure 4b and Table 1). The total *V_oc_* loss consists of radiative recombination loss and non-radiative recombination loss (summarized in Table 1). The MoO*_x_*-passivated device exhibits a higher radiative recombination loss (41.7 mV) and non-radiative recombination loss (217.1 mV) compared to those of the reference CIGS (38.6 mV and 211.3 mV), respectively. Given that the absorber layer fabrication process remained identical except for the introduction of the MoO*_x_* interlayer, the observed increase in voltage losses can be attributed to the presence of the MoO*_x_* layer.

When the incident photon energy is higher than the bandgap of semiconductor materials, electrons can be excited to the conduction band, leaving the holes in the valence band. Photogenerated electrons recombine with holes and release energy in the form of a radiative photon. The introduction of the MoO*_x_* layer suppressed the reaction between S and the Mo back electrode, which forms a wider bandgap CIGS absorber with a high S concentration, leading to partial substitution of Se or passivation of selenium vacancies (Figure 3b). The incorporation of S contributes to a reduction in the defect state density within CIGS, reducing lattice disorder and improving crystalline quality (smaller *E_U_* in Figure 3c). Consequently, the MoO*_x_*-passivated CIGS exhibits a higher radiative recombination voltage, accompanied by a slight increase in radiative recombination voltage loss.

Non-radiative recombination voltage losses typically originate from bulk defects, interface or surface defects, and Auger recombination [18]. When solar cells are subject to low-injection conditions, the Auger recombination can be negligible [18]. The MoO*_x_*-passivated CIGS exhibits a slight reduction in crystallinity (wider FWHM in Figure 1b), diode ideality factor (larger *n* value in Figure 2c), and Urbach energy (larger *E_U_* in Figure 3c); the non-radiative recombination voltage loss of the MoO*_x_*-passivated CIGS is higher than that of the reference CIGS. This phenomenon may be attributed to the introduction of a thick MoO*_x_* layer (20 nm). It is reported that the work function and electrical conductivity of MoO*_x_* are strongly dependent on its thickness, which affects the hole extraction efficiency at the rear interface [10]. A thinner MoO*_x_* passivation layer can achieve good conductivity and form a beneficial spike-like band alignment between the CIGS absorber and MoO*_x_*, resulting in superior photovoltaic performance [10]. Therefore, further optimization of the MoO*_x_* layer thickness should be systematically researched for the improvement of the overall optoelectronic performance.

Given the fundamental differences in the Shockley–Queisser (SQ) limit efficiency and corresponding PV parameters across solar cells with different bandgaps, a direct performance comparison without accounting for the bandgap is not reasonable. Therefore, to objectively evaluate solar cell performance, it is necessary to normalize various parameters against their corresponding bandgap-specific SQ limits [19]. As shown in Figure 4c, the figures of merit for these parameters are utilized to analyze the performance losses of solar cells.

The reference CIGS solar cell achieves a power conversion efficiency equivalent to 38% of the SQ theoretical limit for its corresponding bandgap, indicating a 62% loss in efficiency. The efficiency loss originates from the current density loss factor (*F_sc_* = 11%), *FF* loss due to the series and parallel resistance ($F_{FF}^{res}$ = 6%), *FF* loss associated with V_oc_ deficit ($F_{FF}^{Voc}$ = 22%), the non-radiative open-circuit voltage loss factor ($F_{oc}^{nonrad}$ = 20%), and radiative open-circuit voltage loss factor ($F_{oc}^{rad}$ = 3%). In contrast, the MoO*_x_*-passivated CIGS reaches 40% of the SQ limit. This improvement is primarily attributed to a reduction in the FF loss related to the V_oc_ deficit ($F_{FF}^{Voc}$ = 19%). It has been reported that the MoO*_x_* layer can passivate the rear interface of CIGS solar cells, thereby improving PV performance [5,18]. The mechanism involves the suppression of shunt pathways at the rear interface by the MoO*_x_* layer, leading to an enhanced FF. Furthermore, the MoO*_x_*-passivated CIGS device exhibits consistent *F_sc_*, $F_{FF}^{res}$, and $F_{oc}^{nonrad}$ with the reference CIGS device, indicating that the introduction of the MoO*_x_* passivation layer does not impede the charge carrier extraction. 

**Figure 5:**
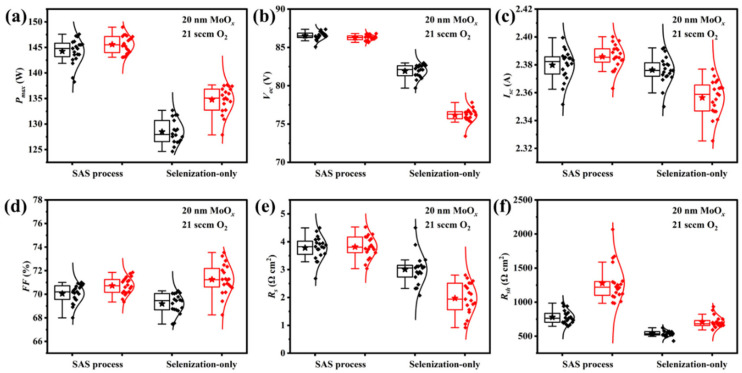
Statistical distributions of photovoltaic parameters for CIGS solar cells fabricated from sulfurization after selenization and selenization-only (without (black box) and with (red box) the MoO*_x_* rear interface passivation layer): (**a**) the maximum output power (*P_max_*), (**b**) open-circuit voltage (*V_oc_*), (**c**) short-circuit current (*I_sc_*), (**d**) fill factor (*FF*), (**e**) series resistance (*R_s_*), and (**f**) shunt resistance (*R_sh_*).

Annealing processes (selenization only or SAS process) would affect the PV performance of CIGS modules without/with a MoO*_x_* passivation layer. The metallic precursor stack without/with a MoO*_x_* passivation layer was subject to selenization only or SAS treatment to evaluate the effect of annealing processes. Regardless of the presence of a MoO*_x_* interface passivation layer, CIGS devices fabricated via the SAS process exhibit a higher maximum output power (*P_max_*) compared to those processed with selenization only (Figure 5). This enhancement is primarily ascribed to the increased *V_oc_* and short-circuit current (*I_sc_*) achieved while maintaining a comparable *FF* after SAS treatment (Figure 5b–d). The SAS-treated CIGS solar cells display an increase in *R_s_* (Figure 5e) and *R_sh_* (Figure 5f). A larger *R_sh_* effectively suppresses shunt recombination losses [18], ultimately leading to superior photovoltaic performance. When the metallic precursor stack without/with a MoO*_x_* passivation layer was subject to selenization only, a higher average *P_max_* was observed from the MoO*_x_*-passivated CIGS. Larger Pmax can be attributed to the enhancement in *FF* and *R_sh_* (Figure 5d,f) as well as the reduction of *R_s_* (Figure 5e). It is indicated that no matter whether the absorbers are fabricated from selenization or SAS treatment, the MoO*_x_* layer can passivate the shunt recombination pathway for the improvement in the *P_max_* of CIGS solar cells. However, lower *V_oc_* and *I_sc_* are observed from the MoO*_x_*-passivated CIGS solar cells. Therefore, the SAS treatment for the metallic stack is beneficial for better PV performance in the CIGS module.

**Figure 6:**
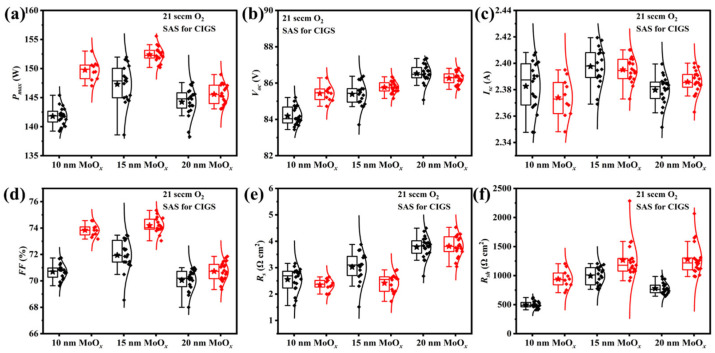
Statistical distributions of photovoltaic parameters for CIGS solar cells with various MoO*_x_* thicknesses (without (black box) and with (red box) the MoO*_x_* passivation layer): (**a**) the maximum output power (*P_max_*), (**b**) open-circuit voltage (*V_oc_*), (**c**) short-circuit current (*I_sc_*), (**d**) fill factor (*FF*), (**e**) series resistance (*R_s_*), and (**f**) shunt resistance (*R_sh_*).

The thickness of the MoO*_x_* layer significantly influences its work function and electrical conductivity [20]. Therefore, optimizing the MoO*_x_* thickness is crucial for enhancing the rear interface properties and process reproducibility of CIGS solar cells. Experimental results (as shown in Figure 6) demonstrate that the introduction of a MoO*_x_* rear passivation layer systematically enhances the *P_max_*, *V_oc_*, and *FF* of CIGS modules. *I_sc_* values initially increase and reach a maximum value at 15 nm, then decrease as the MoO*_x_* thickness is further increased. Nevertheless, the *I_sc_* of the device with a 20 nm MoO*_x_* layer remains higher than that of the device modified with a 10 nm layer. CIGS modules with 10 nm and 20 nm MoO*_x_* passivation layers exhibit *R_s_* values comparable to the corresponding reference samples. When a 15 nm MoO*_x_* layer was introduced in the rear interface of the CIGS modules, a lower average *R_s_* was observed. Furthermore, the MoO*_x_*-passivated CIGS modules show higher *R_sh_* values compared to the corresponding reference samples. These results indicate that the MoO*_x_* layer not only hinders the formation of the MoSe_2_ layer and passivates the rear interface but also effectively suppresses interfacial shunt paths, thereby enhancing the device’s *R_sh_* and comprehensively optimizing the optoelectronic performance.

The oxygen (O_2_) flow rate during sputter deposition serves as a critical parameter for modulating the electrical resistivity of MoO*_x_* thin films, thereby directly influencing their carrier transport behavior within the device [8]. Based on the previously established thickness optimization results, a MoO*_x_* layer thickness of 15 nm was selected for this study to systematically investigate the effect of MoO*_x_* layers—synthesized via reactive sputtering under different O_2_ flow rates—on the performance of CIGS solar cells. To mitigate variations carried out in different selenization batches, all MoO*_x_*-passivated samples prepared under various O_2_ flow rates, along with their corresponding reference samples, were fabricated using identical sputtering and sulfurization-after-selenization (SAS) process conditions.

**Figure 7:**
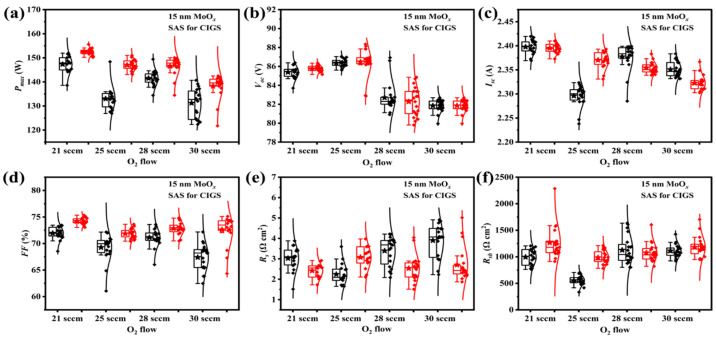
Statistical distributions of photovoltaic parameters for CIGS solar cells with a 15 nm MoO*_x_* rear interface modification layer fabricated in various O_2_ flows (without (black box) and with (red box) the MoO*_x_* passivation layer): (**a**) the maximum output power (*P_max_*), (**b**) open-circuit voltage (*V_oc_*), (**c**) short-circuit current density (*I_sc_*), (**d**) fill factor (*FF*), (**e**) series resistance (*R_s_*), and (**f**) shunt resistance (*R_sh_*).

No matter whether the MoO*_x_* passivation layer is deposited at various O_2_ flow rates, MoO*_x_*-passivated CIGS modules exhibit superior *P_max_* compared to the corresponding reference samples (Figure 7). The enhancement in *P_max_* is attributed to the effective suppression of the reaction between Se and the Mo back electrode by the MoO*_x_* layer (larger *FF* in Figure 7d), which promotes the formation of an ohmic contact at the rear interface and consequently optimizes carrier transport [19]. Additionally, the MoO*_x_* layer increases the *R_sh_* (Figure 7f), leading to the suppression of the shunt losses. However, when the MoO*_x_* layer is sputtered at a high O_2_ flow rate during sputtering (O_2_ flow ≥ 28 sccm), the formation of highly resistive MoO_3_ is favored over MoO*_x_* with suitable conductivity [8]. The high-resistance MoO_3_ layer impedes carrier extraction, resulting in the deterioration of device performance.

Based on these systematic research results, the CIGS solar cell fabricated on a 15 nm-thick MoO*_x_* layer deposited at an O_2_ flow rate of 21 sccm, in combination with the sulfurization-after-selenization (SAS) process, demonstrated the optimum performance. The MoO*_x_*-passivated device achieved an average output power of 152.41 W (corresponding to a substrate size of 1650 mm × 658 mm), with the key photovoltaic parameters as follows: V_oc_ = 85.77 V, I_sc_ = 2.40 A, FF = 74%, and η = 14.0%.

## 4. Conclusions

In this study, a MoO*_x_* rear interface passivation layer was deposited on the surface of the Mo back contact via magnetron sputtering. The MoO*_x_* layer acts as an effective diffusion barrier for the Se and S elements during high-temperature annealing, suppressing the formation of a MoSe_2_ interlayer. This optimization of the interfacial structure reduced void defects and improved interfacial adhesion. The MoO*_x_* passivation layer optimized the rear interface band alignment of the CIGS absorber, facilitating hole extraction and suppressing interfacial recombination. After introducing a thin MoO*_x_* layer, a notable reduction in series resistance and an increase in shunt resistance resulted in an improvement in fill factor (*FF*) and short-circuit current density (*J_sc_*). Although the introduction of MoO*_x_* resulted in slight grain refinement in the CIGS absorber, the beneficial contributions of interface passivation and band alignment optimization were dominant. The best-performing CIGS device was achieved by depositing a 15 nm-thick MoO*_x_* layer at an O_2_ flow rate of 21 sccm, combined with a sulfurization-after-selenization (SAS) post-treatment. This device exhibited an output power of 152.41 W, corresponding to a module *V_oc_* of 85.77 V, *I_sc_* of 2.40 A, *FF* of 74%, and a conversion efficiency (*η*) of approximately 14.0%. Compared to the reference sample, the MoO*_x_*-passivated CIGS achieved an output power increase of ~5 W and an absolute efficiency gain of ~0.5%. These results demonstrated excellent compatibility with existing production lines, offering a viable pathway for enhancing the performance of CIGS thin-film solar cells in an industrial setting.

## Figures and Tables

**Table 1:** Recombination analysis results of CIGS solar cells with/without MoO*_x_* for the rear interface modification layer.

	*E_g_* (eV)	V_oc,SQ_ (mV)	V_oc,rad_ (mV)	*V_oc_* (mV)	Non-Radiative *V_oc_* Losses (mV)	Radiative *V_oc_* Losses (mV)	Total *V_oc_* Losses (mV)
BSL	1.078	837.0	798.4	587.1	211.3	38.6	249.9
MoO*_x_*	1.086	844.5	802.8	585.7	217.1	41.7	258.8

## Data Availability

The original contributions presented in this study are included in the article. Further inquiries can be directed to the corresponding author.
